# Addressing the current challenges in the clinical application of AI-based Radiomics for cancer imaging

**DOI:** 10.3389/fmed.2025.1674397

**Published:** 2025-09-29

**Authors:** Yongzhong Xu, Yunxin Li, Feng Wang, Yafei Zhang, Delong Huang

**Affiliations:** Department of Imaging, Yantaishan Hospital, Yantai, China

**Keywords:** artificial intelligence, Radiomics, cancer imaging, precision medicine, clinical integration, machine learning, diagnostic accuracy

## Abstract

The integration of artificial intelligence (AI) into Radiomics has transformed cancer imaging by enabling advanced predictive modeling, improved diagnostic accuracy, and personalized treatment strategies. However, the clinical application of AI-based Radiomics faces significant challenges that hinder its widespread adoption. Intrinsic limitations, such as limited datasets, data heterogeneity, and the lack of interpretability in AI models, compromise reliability and generalizability. Practical challenges, including integration into rigid clinical workflows, infrastructural constraints, regulatory barriers, and clinician training gaps, further complicate implementation. Addressing these barriers requires coordinated efforts to establish standardized imaging protocols, foster multi-institutional collaborations, and develop centralized repositories of diverse datasets. In addition, challenges programs for healthcare professionals and regulatory reforms are essential to build trust and streamline adoption. Future research should prioritize enhancing AI interpretability, conducting longitudinal studies to assess clinical impact, and incorporating patient-centered approaches to align AI models with precision medicine objectives. By overcoming these challenges, AI-based Radiomics can advance cancer imaging, improve patient outcomes, and contribute to a new era in personalized cancer care.

## 1 Introduction

Radiomics has emerged as a transformative paradigm in medical imaging, providing a novel approach to extracting quantitative data from medical images that goes beyond conventional visual interpretation. This paradigm has been particularly impactful in oncology, where the ability to characterize tumor phenotypes at a refined level has proven invaluable for guiding therapeutic decisions and optimizing patient management (1). Radiomics uses advanced image analysis techniques to extract high-dimensional features that capture the spatial, temporal, and textural characteristics of tumors, offering a comprehensive tumor profile (2). These insights have the potential to significantly improve prognostic assessments, predict treatment responses, and ultimately enhance patient outcomes.

The integration of artificial intelligence (AI) into Radiomics has further revolutionized the field, catalyzing a paradigm shift in the way imaging data is analyzed and interpreted (3–5). AI introduces sophisticated machine learning and deep learning algorithms capable of processing large volumes of complex imaging data with unparalleled precision and efficiency. These algorithms excel at identifying subtle patterns and correlations within imaging datasets that may be imperceptible to human observers. This synergy between AI and Radiomics has propelled advancements in predictive modeling, diagnostic accuracy, and personalized cancer care (6–8). For example, AI-based Radiomics models can predict tumor behavior (9), stratify patients based on risk profiles (10), and identify biomarkers for targeted therapies (11), thereby supporting the broader goals of precision medicine (12).

However, despite these advancements, several challenges continue to delay the full clinical potential of AI-based Radiomics in cancer imaging (13–15). Issues such as data heterogeneity, inconsistencies in imaging protocols, and the lack of large, diverse annotated datasets present significant obstacles. In addition, the clinical adoption of these technologies is often complicated by regulatory barriers, difficulties in validating AI models across diverse patient populations, and the need for seamless integration into existing diagnostic workflows. While the scientific community has made significant progress in exploring the concepts and technological advancements in AI-based Radiomics, most of the current literature does not address in details the practical and clinical challenges that must be resolved to translate these innovations into routine practice.

This mini-review aims to bridge this gap by focusing on the main challenges associated with the clinical application of AI in Radiomics for cancer imaging. It aims to provide a focused discussion on the intrinsic limitations inherent to current studies, as well as the practical barriers that impede real-world implementation. Furthermore, the mini-review provides actionable recommendations for integrating AI and Radiomics into diagnostic workflows, emphasizing strategies to ensure their effective and sustainable use in clinical settings.

## 2 Limitations and challenges

The integration of AI into Radiomics for cancer imaging holds immense promise, yet it faces significant limitations and challenges that need to be addressed to achieve widespread clinical adoption. These obstacles can be broadly categorized into intrinsic limitations inherent to current studies and practical challenges related to clinical implementation. Distinguishing between these two types of barriers is essential, as they require distinct approaches to overcome. Intrinsic limitations primarily stem from methodological issues in research design and data handling, while practical challenges arise from the complexities of integrating AI technologies into real-world healthcare systems. Together, they represent critical barriers that must be resolved to bridge the gap between technological innovation and clinical utility.

### 2.1 Intrinsic limitations of current studies

Intrinsic limitations in AI-Radiomics research are deeply rooted in the methodological, technical, and practical aspects of study design and implementation. These limitations significantly hinder the translation of AI-based Radiomics models into routine clinical practice and compromise their reliability, reproducibility, and generalizability. Addressing these challenges is essential for advancing the field and ensuring the clinical utility of AI-Radiomics technologies.

A significant challenge in AI-Radiomics research is the reliance on small sample sizes and limited datasets (16), often sourced from single institutions or homogeneous patient populations. This restricts the generalizability of AI models across diverse clinical settings and demographics, as specific imaging protocols and scanner types may not represent broader patient groups (17). Consequently, the lack of diversity undermines the robustness of AI algorithms, making them less applicable in real-world scenarios. In addition, small sample sizes reduce statistical power, increase bias and decrease confidence in findings, as models trained on insufficient data may yield unreliable predictions (18). The scarcity of large, annotated datasets is exacerbated by privacy concerns, proprietary restrictions, and non-standardized data formats, underscoring the urgent need for multi-institutional collaborations and centralized imaging repositories to enhance access to diverse, high-quality datasets.

Another significant limitation is the tendency of AI models to overfit when trained on insufficient or narrowly focused data. Overfitting occurs when a model performs exceptionally well on the training dataset but fails to maintain accuracy when applied to new, unseen data (19). This issue is particularly problematic in Radiomics, where the high dimensionality of extracted features increases the risk of overfitting. Models trained on small or homogeneous datasets may learn patterns that are specific to the training data rather than generalizable trends, leading to poor performance in external validation studies (20).

The absence of standardized protocols for feature extraction and data preprocessing further exacerbates this problem. Variability in imaging acquisition methods (such as differences in scanner types, resolution, imaging protocols, and reconstruction algorithms) introduces inconsistencies in the extracted Radiomics features (21). For example, the same tumor imaged on different scanners or using different protocols may yield significantly different Radiomics signatures, complicating the training and validation of AI models (22). In addition, pre-processing parameters and disease characteristics strongly influenced the reproducibility of Radiomics features (23). These inconsistencies highlight the need for standardized imaging protocols and preprocessing workflows to ensure the reproducibility and comparability of Radiomics studies.

The “black-box” nature of many AI models, particularly deep learning algorithms, presents a critical challenge in clinical applications. While these models often achieve high accuracy, their lack of interpretability makes it difficult for clinicians to understand the reasoning behind their predictions (24). This limitation creates skepticism among healthcare professionals, who require clear and evidence-based explanations to inform clinical decision-making. For example, an AI model may predict that a tumor is likely to be aggressive, but without an explanation of the features or patterns driving this prediction, clinicians may be hesitant to rely on the model’s output. The lack of interpretability also complicates the regulatory approval process for AI models, as transparent and explainable algorithms are more likely to gain acceptance from regulatory agencies (25). Techniques such as attention mechanisms, feature importance mapping, and explainable AI frameworks are being explored to address this limitation (26), but their adoption remains limited in current studies. Incorporating interpretability into AI-Radiomics models is essential for building trust among clinicians and ensuring their integration into routine practice.

Many studies fail to incorporate external validation or longitudinal data, which are relevance for assessing the reliability and clinical impact of AI-Radiomics models. External validation involves testing a model on independent datasets that were not used during training, providing a more accurate assessment of its generalizability. However, the lack of diverse and annotated datasets often limits the ability to perform robust external validation, leading to inflated performance metrics that may not translate to real-world settings.

Longitudinal data is equally important for evaluating the long-term reliability and clinical relevance of AI models (27). For example, tracking patient outcomes over time can provide insights into whether AI-driven predictions align with actual disease progression or treatment responses (28, 29). The absence of longitudinal studies in current research limits the ability to assess the true impact of AI-Radiomics on patient care, highlighting the need for studies that go beyond cross-sectional analyses to include temporal dimensions.

In addition to the previously mentioned challenges of limited datasets, data heterogeneity, and interpretability of AI model results, we recognize the importance of explainability and the integration of effective decision support systems in enhancing the clinical utility of AI-based Radiomics. The explainability of AI models is of primary relevance for fostering trust among clinicians and ensuring that AI-driven insights are actionable in clinical settings. Explainability refers to the ability of AI systems to provide understandable and interpretable outputs that clinicians can rely on when making diagnostic or treatment decisions. Without clear explanations of how AI models arrive at their predictions, clinicians may be hesitant to incorporate these tools into their practice. We have elaborated on techniques such as attention mechanisms and feature importance mapping, which can enhance the explainability of AI models. The integration of AI into clinical workflows necessitates the development of robust decision support systems that can effectively translate AI insights into actionable clinical recommendations. These systems should be designed to assist clinicians in interpreting AI outputs and integrating them into their decision-making processes. By providing contextual information and supporting collaborative decision-making, these systems can improve the overall effectiveness of AI in Radiomics.

Additional intrinsic limitations include the lack of consensus on optimal feature selection methods, which can lead to the inclusion of irrelevant or redundant features that dilute model performance. Furthermore, the computational demands of AI-Radiomics models, including the need for high-performance hardware and specialized software, can pose practical challenges for widespread adoption.

### 2.2 Practical challenges for the clinical application of AI in Radiomics for cancer imaging

The clinical application of AI-based Radiomics in cancer imaging faces numerous practical challenges that arise from the complexities of integrating advanced technologies into existing healthcare systems. These challenges span technical, operational, infrastructural, regulatory, and cultural domains, collectively limiting the widespread adoption and effective utilization of AI tools in routine clinical practice. Addressing these issues is essential to unlocking the full potential of AI-Radiomics in improving diagnostic precision, treatment personalization, and patient outcomes.

One of the most pressing practical challenges is the difficulty of incorporating AI tools into established diagnostic workflows, which are often rigid and resistant to change. Traditional healthcare systems are structured around standardized processes and protocols that prioritize consistency and reliability. Introducing AI technologies into these frameworks requires significant adjustments, including the redesign of workflows to accommodate AI-driven insights (30). However, such changes are often met with resistance from clinicians and administrators who may perceive AI tools as disruptive or unnecessary additions to their current practices (31).

Healthcare professionals frequently lack the technical expertise required to operate AI systems effectively, further complicating their integration into clinical workflows (24). The complexity of AI models, coupled with their reliance on advanced data processing techniques, can be intimidating for clinicians who are accustomed to conventional diagnostic tools. This lack of familiarity creates a barrier to adoption, as clinicians may struggle to trust or use AI-driven tools in their practice (32). To address this, comprehensive training programs must be developed to equip healthcare professionals with the knowledge and skills needed to interact with AI systems confidently (33).

Cultural and organizational resistance within healthcare systems further complicates the adoption of AI-based Radiomics. Many clinicians and administrators are skeptical of AI technologies, viewing them as experimental or unreliable tools that lack the evidence base required for widespread use (34). This skepticism is often fueled by the “black-box” nature of AI models, which makes it difficult to understand the reasoning behind their predictions. Clinicians, who rely on transparent and evidence-based approaches to decision-making, may be reluctant to trust AI tools that cannot provide clear explanations for their outputs (35). Fostering a cultural shift toward embracing technology-driven approaches to care is essential for building trust and acceptance among clinicians.

Organizational resistance may also stem from concerns about workflow disruption, increased workload, and the potential for errors or inaccuracies in AI-driven diagnostics (36). Institutions may be hesitant to invest in AI technologies if they perceive them as adding complexity rather than streamlining processes (37). Addressing these concerns requires the development of user-friendly AI systems that integrate seamlessly into existing workflows, minimizing disruption and enhancing efficiency. Furthermore, efforts to educate clinicians and administrators about the benefits and limitations of AI tools can help overcome skepticism and foster acceptance.

The infrastructural requirements of AI-based Radiomics present significant barriers to implementation, particularly in resource-constrained settings. AI models demand high-performance computing resources, including powerful processors, GPUs, and large-scale data storage systems, to handle the computationally intensive tasks of feature extraction, model training, and validation (38). These technologies are expensive and may be inaccessible to smaller healthcare institutions or those operating in low- and middle-income countries (39). The financial burden of acquiring and maintaining such infrastructure can deter institutions from investing in AI tools, regardless of their potential benefits.

Data privacy and security concerns further compound these infrastructural challenges. AI models often rely on large volumes of sensitive patient information, including imaging data, clinical records, and genomic profiles. Ensuring the secure storage, transmission, and processing of this data is critical to maintaining patient confidentiality and compliance with privacy regulations (40). However, achieving this requires robust cybersecurity measures and adherence to complex regulatory frameworks, such as the General Data Protection Regulation (GDPR) in Europe or the Health Insurance Portability and Accountability Act (HIPAA) in the United States. These measures can be resource-intensive and challenging to implement, particularly for institutions with limited technical expertise or funding.

Ethical and legal concerns represent another layer of practical challenges for the clinical application of AI in Radiomics (41, 42). Legal challenges include liability issues, such as determining accountability for errors or adverse outcomes resulting from AI-driven diagnostics. If an AI tool provides an incorrect prediction that leads to a misdiagnosis or inappropriate treatment, questions may arise about whether the responsibility lies with the developer, the clinician, or the institution. Establishing clear legal frameworks for AI use in healthcare is essential for resolving these issues and ensuring the ethical deployment of AI technologies.

Regulatory barriers represent another major practical challenge for the clinical application of AI in Radiomics. AI tools must undergo rigorous validation and approval processes to demonstrate their safety, efficacy, and reliability before they can be used in clinical practice (43). These processes often involve extensive testing across diverse patient populations, ensuring that the models perform consistently and accurately in real-world scenarios (44). However, the lack of standardized guidelines for validating AI models creates uncertainty for developers and healthcare providers, slowing the adoption of these technologies.

The lengthy and unclear nature of regulatory approval processes can discourage innovation and investment in AI-driven diagnostics (45). Developers may face difficulties navigating the complex requirements for clinical trials, data submission, and performance evaluation, while healthcare providers may hesitate to adopt AI tools that have not yet received regulatory approval (46). Additionally, reimbursement policies for AI-driven diagnostics remain poorly defined, creating financial disincentives for healthcare institutions to invest in these technologies. Without clear frameworks for reimbursement, the cost of adopting AI tools may outweigh their perceived benefits, even if they offer significant improvements in diagnostic accuracy and patient outcomes.

Overcoming these practical challenges requires coordinated efforts across multiple domains. Educational initiatives should focus on training healthcare professionals to use AI systems effectively and fostering a cultural shift toward embracing technology-driven approaches to care. Infrastructure development must prioritize the acquisition of high-performance computing resources and the implementation of robust data privacy and security measures. Regulatory reform is needed to streamline validation and approval processes, establish clear reimbursement policies, and encourage innovation in AI-driven diagnostics. Finally, addressing ethical and legal concerns requires the development of transparent, unbiased, and accountable AI models that prioritize fairness and equity in healthcare delivery.

## 3 Implications for future research and clinical practice

The integration of AI and Radiomics into cancer imaging offers transformative potential, but its successful implementation requires deliberate strategies and focused research efforts. Addressing both intrinsic limitations and practical challenges will pave the way for seamless integration into clinical workflows while enhancing the reliability and relevance of AI-Radiomics models. This section outlines key recommendations for integration and highlights priority areas for future research to ensure the widespread adoption and clinical utility of these technologies.

### 3.1 Recommendations for integration

To facilitate the effective integration of AI and Radiomics into diagnostic workflows, several strategies must be prioritized. First, the development of standardized protocols for data collection, preprocessing, and feature extraction is essential. Standardization will mitigate variability in imaging data and ensure consistency across studies, enabling the creation of robust and generalizable AI models (47, 48). Establishing universal guidelines for imaging acquisition and analysis will also enhance reproducibility and comparability of results, fostering trust in AI-driven diagnostics.

However, it is essential to recognize that the integration of diverse data types, such as imaging modalities, clinical data, and genomic information, poses significant challenges. These data types often exhibit inherent variability in formats, scales, and quality, which can hinder the harmonization process. Therefore, a concerted effort is required to develop frameworks that facilitate the integration of heterogeneous data sources. This includes the adoption of common data models and ontologies that can bridge gaps between different datasets, ensuring that AI algorithms can effectively learn from a wide range of inputs.

Multi-institutional collaborations should be encouraged to address the current scarcity of large, diverse datasets (49–51). Institutions can create comprehensive datasets that reflect diverse patient populations and imaging techniques by pooling resources and expertise. Such collaborations will improve the generalizability of AI models and also accelerate the development of clinically relevant tools. Furthermore, initiatives to share annotated datasets and imaging repositories should be supported to reduce redundancy and promote innovation in the field.

Training programs for clinicians are another critical component of successful integration (52). Healthcare professionals must be equipped with the knowledge and skills to understand, operate, and interpret AI-driven tools. Educational initiatives should focus on demystifying AI technologies, emphasizing their applications in Radiomics, and providing practical guidance on their use in clinical workflows (53, 54). These programs will help build trust and confidence in AI systems, fostering a culture of collaboration between clinicians and technology developers.

### 3.2 Future research directions

Future research should focus on addressing gaps that currently limit the clinical adoption and impact of AI-Radiomics. One key area is the exploration of AI interpretability (55). Developing models that provide transparent and explainable outputs will enhance trust among clinicians, enabling them to understand the rationale behind AI-driven decisions (56). Research into techniques such as attention mechanisms and feature importance mapping can help make AI systems more accessible and trustworthy (57).

Longitudinal studies are also urgently needed to evaluate the long-term impact of AI-Radiomics on patient outcomes (58). By tracking patients over time, researchers can assess how AI-driven insights influence treatment decisions, disease progression, and overall survival rates (59, 60). Such studies will provide critical evidence of the clinical utility of AI technologies, supporting their adoption in routine practice.

In addition, patient-centered approaches should be investigated to enhance the relevance of AI findings. This includes exploring ways to incorporate patient-specific factors, such as genetic profiles and lifestyle data, into Radiomics analyses (61). Tailoring AI models to individual patients enhances diagnostic accuracy and enables personalized treatment strategies, effectively aligning with the broader objectives of precision medicine.

### 3.3 Framework for implementation

In this subsection, we summarize the key actionable recommendations to enhance the integration of AI-based Radiomics into clinical practice. These recommendations aim to address the challenges identified in previous sections and facilitate the effective application of AI technologies in cancer imaging. To facilitate the effective integration of AI and Radiomics into diagnostic workflows, we present a summary of the current state of the art and actionable recommendations in Table 1.

**Table 1:** Summary of current challenges and recommended actions for integrating AI-based Radiomics in clinical practice.

State of the art	To-do actions
Limited datasets and data heterogeneity	Establish multi-institutional collaborations for dataset sharing and creation of centralized repositories.
Lack of interpretability in AI models	Develop and implement explainable AI frameworks and techniques.
Rigid clinical workflows	Redesign workflows to integrate AI tools seamlessly and provide training for clinicians.
Regulatory barriers and lengthy approval processes	Advocate for regulatory reforms to streamline approval processes and establish clear reimbursement policies.
Insufficient clinician training on AI technologies	Implement comprehensive training programs for healthcare professionals on AI tools and their applications in Radiomics.
Absence of standardized protocols for data handling	Develop universal guidelines for imaging acquisition, data preprocessing, and feature extraction.
Need for longitudinal studies to assess clinical impact	Conduct longitudinal studies tracking patient outcomes to evaluate AI’s effectiveness over time.

## 4 Concluding remarks

This mini review highlights the transformative potential of AI and Radiomics in cancer imaging while discussing the intrinsic limitations of current studies, alongside practical challenges like integration into clinical workflows, clinician training, and regulatory barriers. Overcoming these challenges will require methodological advancements, multi-institutional collaborations, standardized protocols, and education-driven efforts to ensure seamless adoption. Future research should focus on enhancing AI interpretability, conducting longitudinal studies, and adopting patient-centered approaches to ensure clinical relevance. Addressing these challenges will advance cancer imaging, improve diagnostic accuracy, and contribute to precision medicine, which requires a collective effort to bridge the gap between innovation and clinical application for a new era in cancer care.
